# Percutaneous Cryoablation Under Local Anesthesia for Pulmonary Metastases From Colorectal Cancer: Long‐Term Outcomes From a Single‐Institution Retrospective Cohort

**DOI:** 10.1002/cnr2.70550

**Published:** 2026-04-19

**Authors:** Shun Yorimori, Kaoru Kaseda, Yusuke Aoki, Kosuke Sugino, Takahiro Suzuki, Yu Okubo, Shigeki Suzuki, Kyohei Masai, Masashi Tamura, Masanori Inoue, Hideki Yashiro, Seishi Nakatsuka, Yoshikane Yamauchi, Yotaro Izumi, Masafumi Kawamura, Masahiro Jinzaki, Keisuke Asakura

**Affiliations:** ^1^ Division of Thoracic Surgery, Department of Surgery Keio University School of Medicine Tokyo Japan; ^2^ Department of Radiology Keio University School of Medicine Tokyo Japan; ^3^ Department of Radiology Fujita Health University School of Medicine Toyoake Japan; ^4^ Department of Radiology Hiratsuka City Hospital Hiratsuka Japan; ^5^ Department of Surgery Teikyo University School of Medicine Tokyo Japan

**Keywords:** local treatment, percutaneous cryoablation, pulmonary metastases from colorectal cancer

## Abstract

**Background:**

Surgical resection is the standard treatment for pulmonary metastases from colorectal cancer, but its safety and long‐term efficacy in medically inoperable patients remain limited. Percutaneous cryoablation is a minimally invasive alternative to surgical resection and has gained increasing attention in recent years.

**Aims:**

This retrospective study aimed to evaluate complication rates, local tumor control, and overall survival associated with percutaneous cryoablation for pulmonary metastases from colorectal cancer.

**Methods and Results:**

We retrospectively reviewed patients treated with percutaneous cryoablation from 2002–2017. Complications were defined as adverse events ≥ grade 2 per Common Terminology Criteria for Adverse Events version 5.0. In total, 126 metastatic pulmonary tumors in 48 patients were treated across 73 sessions. Complications occurred in 18 sessions (24.7%), including 2 grade ≥ 3 events (2.7%). No treatment‐related deaths occurred within 30 days. With a median follow‐up of 9.7 months (maximum, 155.4 months), local tumor control rates at 1, 3, and 5 years were 74.5%, 58.8%, and 56.4%, respectively. Median overall survival was 3.8 years, with 1‐, 3‐, 5‐, and 10‐years rates of 86.8%, 60.4%, 41.9%, and 36.6%, respectively. The relatively short median follow‐up should be considered when interpreting these long‐term local control estimates. Six patients achieved 10‐years survival.

**Conclusion:**

Percutaneous cryoablation is a safe, minimally invasive option that provides favorable local tumor control and encouraging long‐term survival outcomes.

## 1 Introduction

Colorectal cancer is one of the most common and lethal malignancies worldwide, with lung being the second most frequent site of distant metastasis after the liver metastasis [1, 2, 3]. Surgical resection remains the standard treatment for resectable pulmonary metastases, and multiple studies have demonstrated that complete resection is associated with favorable long‐term survival [4, 5, 6]. However, surgery is not feasible for many patients due to impaired cardiopulmonary function, comorbidities, or prior thoracic surgery. For medically inoperable or unresectable cases, local treatment modalities such as stereotactic body radiation therapy (SBRT), radiofrequency ablation (RFA), and microwave ablation (MWA) are used as alternative options [7, 8, 9, 10, 11, 12, 13, 14, 15].

In recent years, the concept of oligometastasis has attracted increasing attention. This condition is defined by a limited number of metastatic lesions, typically five or fewer. In colorectal cancer, multiple studies have reported that local therapies for oligometastatic disease—such as surgical resection and radiation therapy—may contribute to prolonged survival [16, 17]. Consequently, the role of local treatment for pulmonary metastases from colorectal cancer is being actively reevaluated.

Percutaneous cryoablation has emerged as a promising minimally invasive local treatment due to its ability to provide real‐time visualization of the ablation zone and safely treat central lesions or those adjacent to critical structures [18, 19, 20, 21]. Since 2002, our institution has employed cryoablation for thoracic malignancies, including primary lung cancer and pulmonary metastases, with multiple reports demonstrating its efficacy and safety [22, 23, 24, 25, 26, 27, 28, 29]. Although several small‐scale studies have described the use of cryoablation for pulmonary metastases from colorectal cancer, evidence regarding long‐term outcomes—particularly beyond 5 years—remains limited [30, 31, 32]. Therefore, we retrospectively evaluated the safety, local tumor control, and long‐term outcomes of percutaneous cryoablation for pulmonary metastases originating from colorectal cancer.

## 2 Methods

### 2.1 Ethics

This retrospective cohort study was conducted at a single institution in accordance with the principles of the Declaration of Helsinki (revised in 2013) and received approval from the Ethics Committee of Keio University School of Medicine in 2021 (approval ID: 20210003). Owing to the retrospective design, the requirement for informed consent was waived.

### 2.2 Patients

This study included consecutive patients who underwent percutaneous cryoablation for pulmonary metastases from colorectal cancer at our institution between July 2002 and September 2017.

The pretreatment criteria for percutaneous cryoablation at our institution were as follows: (i) surgical resection was deemed unsuitable due to factors such as prior thoracic surgery, multiple pulmonary tumors, patient refusal, impaired pulmonary function, comorbidities, or advanced age; (ii) absence of untreated extrapulmonary malignancies; (iii) an Eastern Cooperative Oncology Group (ECOG) performance status of 0 or 1; (iv) platelet count ≥ 50 000/μL; (v) international normalized ratio of < 1.5; (vi) estimated life expectancy of ≥ 12 months; and (vii) provision of written informed consent.

### 2.3 Cryoablation Technique

The cryoablation technique was previously described [23, 26, 28, 29]. The procedures were performed in a computed tomography (CT) suite under local anesthesia by two interventional radiologists or thoracic surgeons. The chief interventionist (S.N.), who had over 30 years of experience in ablation treatment, performed all 126 cryoablation sessions in this study.

Cryoablation was conducted using a multidetector CT scanner (Aquilion 64; Toshiba Medical Co. Ltd., Tokyo, Japan). Continuous electrocardiogram monitoring was performed during the procedure. After skin disinfection with iodine, local anesthesia was administered via subcutaneous injection of 10–20 mL of 1% lidocaine from the skin to the pleura. Approximately 20 min before the procedure, 15 mg of pentazocine was administered via intramuscular injection. The appropriate patient position on the CT table was determined according to the location of the target lesion.

A 21‐gauge guiding needle was inserted into the target lesion under three‐slice CT fluoroscopic guidance. A customized 8‐ or 11‐gauge coaxial stainless steel introducer (Silux, Kawaguchi, Japan), composed of an inner guiding sheath and an outer sheath, was advanced along the guiding needle. After removal of the inner sheath and guiding needle, a cryoprobe (CRYOcare Cryosurgical Unit; Endocare, Irvine, CA, USA; currently distributed by Varian Medical Systems, Palo Alto, CA, USA) with a diameter of 2.4 or 3.0 mm was inserted through the outer sheath.

Cryoablation was performed using a triple freeze–thaw cycle. Before July 2006, freezing durations were 5, 5, and 10 min. After July 2006, the protocol was modified to 5, 10, and 10 min. High‐pressure argon gas was used for freezing, and active thawing with high‐pressure helium gas was applied until the probe temperature reached 20°C. CT imaging was conducted after each cycle to confirm that the ablation zone encompassed the target lesion with a minimum safety margin of 5 mm. The number and size of cryoprobes were selected to ensure adequate coverage based on the tumor diameter.

**FIGURE 1:**
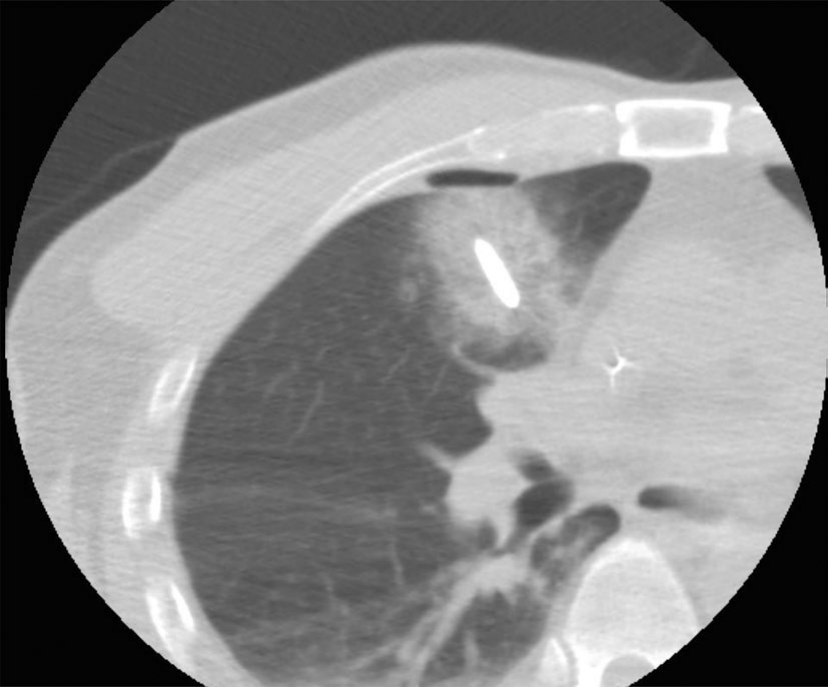
Computed tomography images of percutaneous cryoablation of the tumor located in segment 5 of the right lung.

Following the procedure, fibrin glue (Bolheal; Chemo‐Sero‐Therapeutic Research Institute, Kumamoto, Japan) was injected through the outer sheath to prevent pneumothorax or hemothorax. In cases of substantial pneumothorax (> 40% of the hemithorax), manual aspiration was performed using an 18‐gauge cannula. Immediately after the procedure, a CT scan was conducted to assess the ablation zone and confirm the absence of complications such as intracardiac gas or pneumothorax. Prophylactic intravenous cefotiam hydrochloride (1 g) was administered three times: preoperatively, immediately postoperatively, and on the first postoperative day. Figure 1 shows CT images of percutaneous cryoablation of the tumor located in segment 5 of the right lung.

### 2.4 Follow‐Up and Outcome Measures

To assess procedure‐related complications, chest CT scans were performed the day after percutaneous cryoablation. Additionally, to evaluate treatment response and local control, follow‐up CT scans were performed 1 week and 1 month post‐procedure and subsequently at intervals of 3 to 4 months.

Data were collected from the medical charts of three authors (S.Y., K.K., and K.A.), CT images, and procedural records of cryoablation. Variables were categorized into patient‐related and tumor‐related factors as follows: (1) patient‐related factors: age, sex, ECOG performance status, total number of tumors, total number of cryoablation sessions, smoking history, and prior treatments before cryoablation; (2) tumor‐related factors: maximum tumor diameter, tumor location (central/noncentral), primary tumor site, and presence of extrathoracic metastases before cryoablation.

A tumor was classified as central if the geometric center of the tumor was located within the inner one‐third of the lung parenchyma on axial CT images. This was determined on the slice showing the maximal tumor diameter by measuring the distance from the mediastinal or hilar side toward the pleural surface. Local tumor progression was defined as an increase in tumor diameter of more than 20% compared to the smallest diameter (nadir) of the ablation zone measured after cryoablation or the appearance of nodular enhancement [19, 20]. The assessment was performed by multiple board‐certified radiologists at our institution.

Overall survival (OS) was defined as the period from the initial percutaneous cryoablation to death or the date of the last follow‐up. Primary local tumor control was defined as the interval from the initial cryoablation to the first radiological evidence of local recurrence; in the absence of local recurrence, the interval was measured until the last imaging evaluation. In cases where local recurrence was observed and patients underwent repeated cryoablation for the same lesion, overall local tumor control was defined as the interval from the initial cryoablation to the detection of recurrence following the final cryoablation session.

Adverse events were defined as treatment‐related complications occurring within 30 days post‐treatment and classified as grade 2 or higher according to Common Terminology Criteria for Adverse Events (CTCAE) version 5.0, based on adjudication by multiple experienced thoracic surgeons and radiologists at our institution. Any death within 30 days post‐treatment was defined as 30‐days mortality.

### 2.5 Statistical Analysis

The Kaplan–Meier method was used to estimate primary and overall local tumor control rates and OS. Potential prognostic factors associated with local tumor progression were analyzed using the log‐rank test, the univariable Cox regression, and the multivariable Cox regression. *p*‐values < 0.05 were considered statistically significant. The statistical analyses were performed using the Statistical Package for the Social Sciences (SPSS) software (version 30; IBM Corp., Armonk, NY, USA).

## 3 Results

### 3.1 Patients and Tumor Characteristics

The characteristics of the patients and tumors are summarized in Table 1. A total of 48 patients underwent percutaneous cryoablation, during which 126 tumors were treated across 73 sessions. The median age was 67 years (range, 32–88), and 32 patients (66.7%) were male. An ECOG performance status of 0 was observed in 38 patients (79.2%), and 21 patients (43.8%) had a history of smoking.

**TABLE 1:** Characteristics of patients and tumors treated with cryoablation (*n* = 48, tumors = 126).

Characteristics	Value
Age (median, range)	67, 32–88
Sex	
Male	32 (66.7%)
Female	16 (33.3%)
ECOG performance status	
0	38 (79.2%)
1	10 (20.8%)
Charlson comorbidity index (median, range)	6, 6–11
Pulmonary function	
VC, L (median, range)	2.8 (1.6–4.6)
% VC, % (median, range)	87 (49–116)
FEV1.0, L (median, range)	2.0 (1.0–3.5)
FEV1.0%, % (median, range)	77 (53–95)
Primary reason for surgical unsuitability	
Prior treatment (surgery or radiation therapy)	18 (37.5%)
Multiple pulmonary tumors	14 (29.2%)
Impaired pulmonary function	5 (10.4%)
Patient refusal	5 (10.4%)
Severe comorbidities	4 (8.3%)
Poor performance status	2 (4.2%)
Number of tumors targeted per patient	
Median, range	2, 1–11
1	17 (35.4%)
2	16 (33.3%)
3	4 (8.3%)
≥ 4	11 (22.9%)
Total number of cryoablation sessions per patient	
Median, range	1, 1–6
1	31 (64.6%)
2	13 (27.1%)
3	3 (6.3%)
6	1 (2.1%)
Smoking status	
Nonsmoker	27 (56.2%)
Current or former smoker	21 (43.8%)
Treatment history for pulmonary metastases prior to cryoablation (overlapped)	
Chemotherapy	18 (31.6%)
Surgery	23 (40.4%)
Radiotherapy	6 (10.5%)
Absent	10 (17.5%)
Site of extrathoracic metastases before cryoablation	
Liver	9 (18.7%)
Prostate	1 (2.1%)
Primary site	
Colon	28 (58.3%)
Rectum	20 (41.7%)
Tumor diameter, cm	
Median, range	1.1, 0.3–5.8
< 1	48 (38.1%)
1 ≤, < 2	53 (41.4%)
2 ≤, < 3	20 (15.6%)
3 ≤	5 (4.9%)
Tumor location	
Central	27 (21.4%)
Noncentral	99 (78.6%)

*Note:* Values are presented as *n* (%).

The median number of tumors treated per patient was 2 (range, 1–11), and the median number of cryoablation sessions per patient was 1 (range, 1–6). Before cryoablation, 18 patients (37.5%) had received chemotherapy, 23 (47.9%) had undergone surgical resection, and 6 (12.5%) had received radiotherapy. Thirty six patients had received prior treatment before cryoablation. Extrapulmonary metastases were present in 10 patients (20.8%), including liver metastases in 9 patients (18.7%) and prostate metastasis in 1 patient (2.1%). The primary tumor site was the rectum in 20 patients (41.7%) and the colon in 28 patients (58.3%). The median tumor diameter was 1.1 cm (range, 0.3–5.8). Among the 126 treated tumors, 27 tumors (21.4%) were classified as central lesions. We summarized tumor characteristics by centrality in a Table S1. No significant differences were observed between central and noncentral tumors in tumor diameter, lobar distribution, or the number of concurrently treated tumors.

### 3.2 Procedural Safety

The post‐procedural outcomes are summarized in Table 2. The median post‐cryoablation hospitalization duration was 2 days (range, 2–13), with discharge occurring on postoperative day 2 in 51 sessions (69.8%). Of the 73 sessions, adverse events were recorded in 18 (24.7%), of which 16 (21.9%) were CTCAE grade 2 and 2 (2.7%) CTCAE grade 3. Table 3 summarizes the adverse events of cryoablation. Pneumothorax was the most common complication (17 sessions, 23.3%), and chest tube drainage was required in 11 sessions (17.8%). Empyema was observed in one patient (1.4%). No deaths occurred within 30 days.

**TABLE 2:** Post‐treatment outcomes following cryoablation (*n* = 73).

Characteristics	Value
Duration of post‐cryoablation hospitalization, day	
Median, range	2, 2–13
Adverse events (grade 2 or higher)	
Grade 2	16 (22.0%)
Grade 3 or higher	2 (2.7%)
Absent	55 (75.3%)
30‐days mortality	0 (0.0%)

*Note:* Values are presented as *n* (%) except for duration of post‐cryoablation hospitalization.

**TABLE 3:** Adverse events of the cryoablation procedure (*n* = 73).

Characteristics	Value
Pneumothorax	17 (23.3%)
Chest tube insertion	11 (15.1%)
Manual aspiration	4 (5.5%)
Chest tube insertion + pleurodesis	2 (2.7%)
Empyema	1 (1.4%)

*Note:* Values are presented as *n* (%).

### 3.3 Local Tumor Control and Overall Survival

**FIGURE 2:**
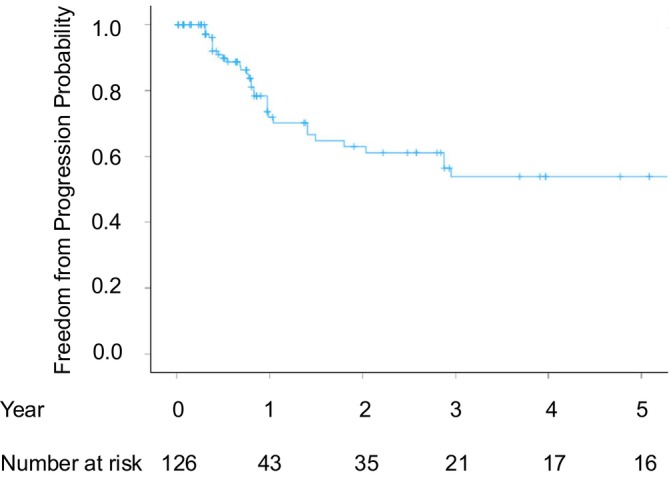
Primary local tumor control rates for all the treated tumors.

**FIGURE 3:**
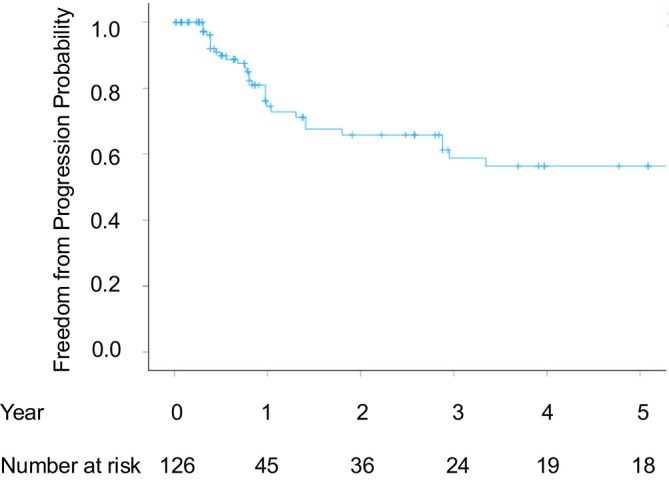
Overall local tumor control rates for all the treated tumors.

The median follow‐up duration was 9.7 months (range, 0.2–186.4), and the distribution of follow‐up duration is shown in Table 4. Local recurrence was observed in 32 tumors (25.4%) among the 126 pulmonary metastases treated with initial cryoablation. The primary local tumor control rates were 71.9% at 1 year, 53.9% at 3 years, and 53.9% at 5 years post‐initial treatment (Figure 2). Among the recurrent tumors, four tumors in three patients underwent repeat cryoablation. One tumor recurred 1.5 years after the initial procedure and was treated with a second cryoablation, after which no recurrence was observed for 3.2 years. The remaining three recurred at 0.6, 2.5, and 3.8 years following the second cryoablation. The first two tumors were subsequently treated with surgical resection, and the other, with SBRT, all resulting in favorable local control. All three patients who underwent repeat cryoablation achieved long‐term survival exceeding 10 years. In contrast, when recurrence after cryoablation was not amenable to further local therapy, most patients subsequently received systemic chemotherapy or best supportive care. The overall local tumor control rates were 74.5% at 1 year, 58.8% at 3 years, and 56.4% at 5 years post‐initial treatment (Figure 3). Among the 126 treated tumors, 17 remained free of recurrence for over 5 years, and eight tumors remained recurrence‐free for over 10 years.

**TABLE 4:** Distribution of follow‐up duration (*n* = 126).

Characteristics	Value
Distribution of follow‐up duration, months	
Median, range	9.7, 0.2–186.4
< 6	42 (33.3%)
6 ≤, < 12	38 (30.2%)
12 ≤, < 36	22 (17.5%)
36 ≤, < 60	6 (12.5%)
60 ≤	18 (14.3%)

*Note:* Values are presented as *n* (%).

**FIGURE 4:**
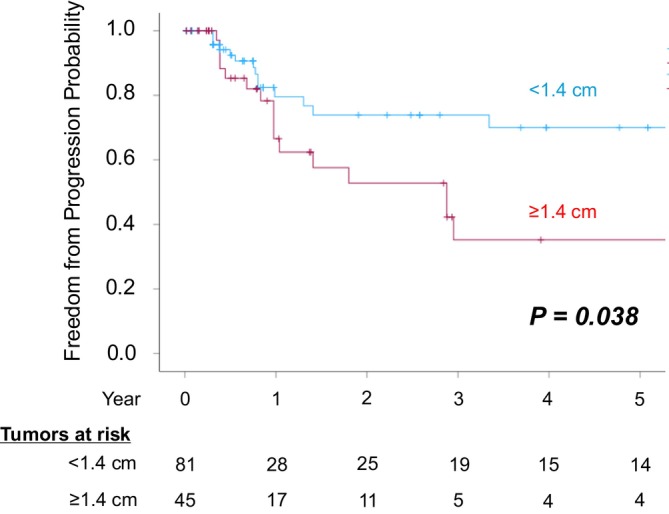
Overall local tumor control rates for all the treated tumors according to tumor diameter.

**FIGURE 5:**
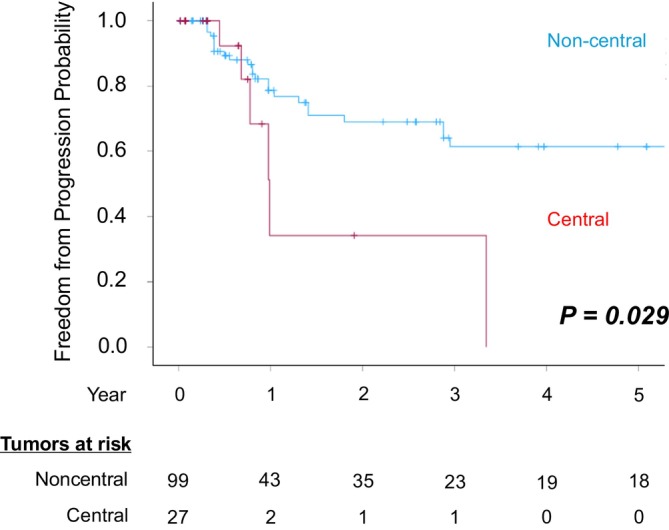
Overall local tumor control rates for all the treated tumors according to tumor location.

**FIGURE 6:**
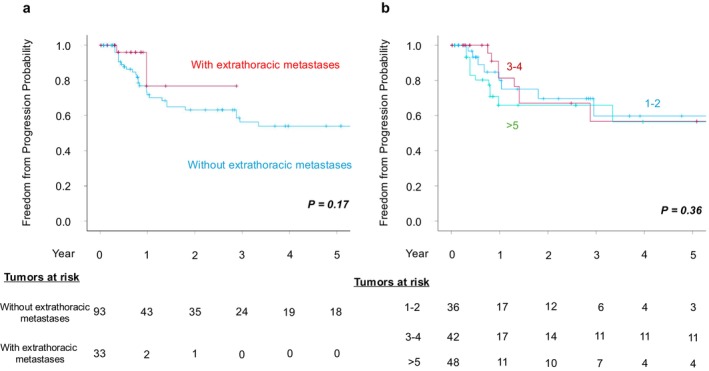
(a) Overall local tumor control rates for all the treated tumors based on extrathoracic metastases before cryoablation. (b) Overall local tumor control rates for all the treated tumors based on the number of concurrent tumors treated.

Receiver operating characteristic (ROC) curve analysis identified a cutoff value of 1.4 cm for tumor diameter (Figure S1). In the Kaplan–Meier analysis stratified by this threshold, patients with tumors ≥ 1.4 cm demonstrated significantly lower overall local tumor control compared with those with tumors < 1.4 cm (log‐rank test, *p* = 0.038) (Figure 4). In addition, when comparing overall local tumor control rates by tumor location, central tumors showed significantly lower control rates than noncentral tumors (log‐rank test, *p* = 0.029) (Figure 5). Moreover, neither the presence of extrathoracic metastases before treatment nor the number of concurrent tumors treated was associated with overall local tumor control rates (log‐rank test, *p* = 0.17, *p =* 0.36)(Figure 6a,b). To identify risk factors for local recurrence, we performed both univariable and multivariable analyses, and the results are presented in Table 5. Tumors ≥ 1.4 cm (HR = 2.13; 95% Cl 1.05–4.32; *p* = 0.037) and central tumor location (HR = 2.77; 95% Cl 1.10–6.95; *p* = 0.03) were identified as significant risk factors for local tumor progression.

**TABLE 5:** Risk factors for local tumor progression.

Variable	Univariate analysis		Multivariable analysis	
	Hazard ratio (95% CI)	*p*	Hazard ratio (95% CI)	*p*
Tumors ≥ 1.4 cm (vs < 1.4 cm)	2.07 (1.02–4.20)	0.043	2.13 (1.05–4.32)	0.037
Central (vs. noncentral)	2.64 (1.06–6.56)	0.037	2.77 (1.10–6.95)	0.03
Right lung (vs. left lung)	0.49 (0.23–1.04)	0.061		
Rectum (vs. Colon)	0.69 (0.33–1.42)	0.31		
Site of extrathoracic metastases before cryoablation	0.38 (0.09–1.62)	0.19		
The number of concurrently treated tumors	1.67 (0.82–3.42)	0.16		

Primary local tumor control rates were analyzed using the same approach. Tumors ≥ 1.4 cm showed a trend toward lower control rates compared with tumors < 1.4 cm. However, this difference did not reach statistical significance (log‐rank test, *p* = 0.084) (Figure S2). On the other hand, central tumors exhibited significantly lower primary local tumor control rates than did noncentral tumors (log‐rank test, *p* = 0.018) (Figure S3). Consistent with the overall analysis, neither the presence of extrathoracic metastases nor the number of tumors treated concurrently was associated with primary local tumor control rates (log‐rank test, *p* = 0.12, *p =* 0.38) (Figure S4).

**FIGURE 7:**
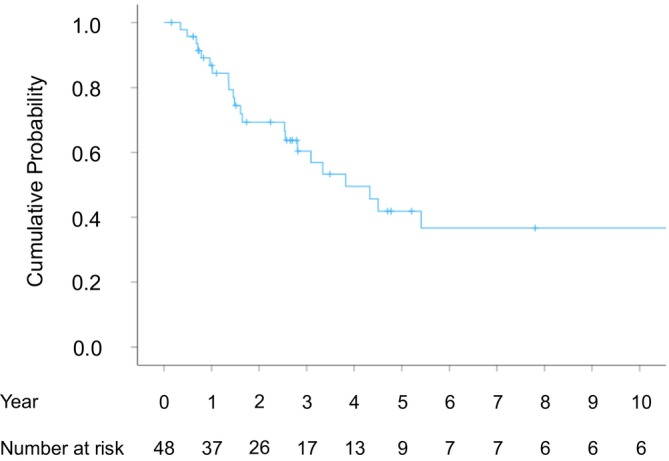
Overall survival rates for all the patients.

The median OS was 3.8 years (range, 2.2–5.4 years), with overall survival rates of 86.8% at 1 year, 60.4% at 3 years, 41.9% at 5 years, and 36.6% at 10 years post‐initial treatment (Figure 7). Among the 48 patients, nine survived for over 5 years, and five survived for over 10 years.

## 4 Discussion

In a study of percutaneous cryoablation for pulmonary malignancies, Callstrom reported grade 3 complications in 4.7% of cases and pneumothorax requiring chest tube drainage in 23.0% of procedures [19] Similarly, Baère reported grade 3 complications in 6.0% of cases and pneumothorax requiring drainage in 18.8% [20]. McDevitt documented a 15.0% rate of pneumothorax requiring drainage [21]. In addition, Izaaryene reported that among patients with peripheral pulmonary metastases from colorectal cancer treated with cryoablation, grade 3 complications occurred in 10% of cases and pneumothorax requiring drainage in 34% [32]. Compared with these prior findings, the complication rates in our study were lower, suggesting that the procedure was performed with a favorable safety profile.

In SBRT, grade 3 complications were reported in 1.5%–6.4% of cases [13, 33, 34]. In contrast, RFA was associated with grade 3 complications in 2.4%–3.0% of cases and with pneumothorax requiring drainage in 14%–25% [14, 15, 35, 36, 37]. When compared with this study and prior studies on cryoablation, these findings appear comparable, supporting the favorable safety profile of cryoablation.

The overall local tumor control rates in the present study were 74.5% at 1 year, 58.8% at 3 years, and 56.4% at 5 years post‐initial treatment. In comparison, Callstrom reported rates of 91.1% at 1 year and 84.4% at 2 years, whereas Baère reported rates of 87.9% at 1 year and 79.2% at 3 years. The slightly lower rates observed in the current study may be attributable to the higher proportion of larger tumors. Prior studies have identified tumors diameter ≥ 2 cm as a significant risk factor for local recurrence [28]. In Callstrom's study, only 6% of tumors measured ≥ 2 cm [19], and in Baère's study, the proportion was 1.7% [20]. In contrast, 20.5% of tumors in the present study were ≥ 2 cm, which may partly explain the lower local control rates. Additionally, in this study, a tumor diameter cutoff value of ≥ 1.4 cm—derived from ROC curve analysis—was identified as a significant risk factor for local recurrence on overall local tumor control analysis. However, the 1.4 cm cutoff identified in this study should be interpreted as an exploratory, supportive indicator rather than an absolute threshold for clinical decision‐making. Consistent with previous reports, these findings further suggest that tumor diameter is an important prognostic factor for local recurrence and that patients with larger tumors may require more careful post‐treatment surveillance. Importantly, in multivariable analysis, tumor diameter ≥ 1.4 cm remained an independent risk factor for local recurrence, indicating that the adverse impact of tumor size was not solely explained by other baseline factors. In contrast, primary local tumor control rates followed a trend similar to overall local tumor control rates, but the differences were not statistically significant. This discrepancy likely reflects the difficulty of securing adequate ablation margins in larger tumors. Such tumors frequently require repeat interventions, and even after retreatment, achieving durable local control is challenging. As a result, differences in local tumor control rates become more pronounced when overall local tumor control rates are evaluated.

In studies restricting treatment to peripheral tumors, the local control rates were 95% at 1 year and 89% at 2, 3, and 4 years, which were superior to the outcomes of the present study and those reported in previous literature [32]. This difference likely reflects the restriction of the treated population to peripheral tumors located within 2 cm of the pleural surface. In addition, definitions of local recurrence differ between studies. Izaaryene defined local recurrence as the appearance of new tumor foci within or in contact with the ablation zone on follow‐up CT [32], whereas in our study, local recurrence was defined as an increase in tumor diameter of more than 20% compared to the smallest diameter measured after cryoablation based on previously published criteria [19, 20]. Because our definition was broader and more sensitive, tumors in our cohort were more likely to be classified as local recurrence. In our cohort, both primary and overall local tumor control rates were significantly lower for centrally located tumors than for noncentral tumors. For central tumors, proximity to major vessels and other critical structures likely attenuates effective cooling and restricts the achievable ablation margins, contributing to inferior control. This interpretation is further supported by our multivariable analysis, in which central tumor location remained independently associated with a higher risk of local recurrence.

According to previous reports, the local control rates of SBRT ranged from 62%–95% at 1 year, 54%–86% at 3 years, and 56%–77% at 5 years [13, 33, 34]. However, SBRT outcomes in pulmonary metastases from colorectal cancer vary substantially according to radiation dose and fractionation schedule, and therefore direct comparison across studies should be made with caution. The local control rates of RFA were 81%–92% at 1 year and 64%–89% at 2 years [14, 36, 37, 38]. When compared with the present study and other prior cryoablation series, these outcomes are similar, suggesting that cryoablation offers adequate therapeutic efficacy.

Cryoablation provides treatment outcomes and safety profiles comparable to those of other local therapies while offering distinct advantages. SBRT is generally difficult to perform repeatedly on the same lesion [39, 40]. In contrast, cryoablation can be applied multiple times to the same lesion, and in the present study, we encountered cases in which local control was subsequently achieved by multiple cryoablations. Moreover, SBRT is often challenging in cases where tumors are widely distributed or located in proximity to major blood vessels or organs [39, 40]. In this study, however, up to seven tumors were successfully treated in a single session. Notably, the number of lesions treated in a single session was not associated with local control. Furthermore, cryoablation was safely performed for central tumors adjacent to major vessels or organs, consistent with prior evidence indicating that cryoablation does not cause damage to blood vessels or bronchi [41]. Similarly, the use of RFA and MWA near critical structures is limited by the risk of thermal injury [41]. By contrast, the current treatment outcomes of cryoablation for central tumors are insufficient, indicating the need for further technical refinements—such as increasing the number of cryoprobes—to improve local control in this population. In addition, cryoablation is reported to cause less intraoperative pain than RFA or MWA owing to the analgesic effect of tissue cooling [42, 43]. In the present study, all the procedures were performed under local anesthesia, suggesting that cryoablation may offer advantages in terms of pain management.

Previous studies on cryoablation for pulmonary metastases from colorectal cancer did not report long‐term local control or survival beyond 5 years. In the present study, however, the overall local control rate at 5 year was 56.4%, with 18 patients remaining recurrence‐free for ≥ 5 years and 8 patients, ≥ 10 years. OS was 60.4% at 5 years and 41.9% at 10 years. Among the 48 treated patients, nine survived for ≥ 5 years and six for ≥ 10 years. Accordingly, by demonstrating long‐term treatment outcomes for pulmonary metastases from colorectal cancer, this study has substantial clinical implications. However, OS should be interpreted with caution because it is strongly influenced by the underlying oncologic course prior to treatment before cryoablation, and subsequent systemic therapies.

This study has several limitations. First, it was a retrospective, single‐center, non‐randomized study. Because treatment allocation was based on clinical judgment rather than randomization, selection bias is possible and may limit the generalizability of our findings. Second, the sample size was limited because of the single‐center design. Nevertheless, to the best of our knowledge, this represents the largest cryoablation study for pulmonary metastases from colorectal cancer to date. Third, the proportion of censored observations was substantial, potentially reducing statistical power and precision of time‐to‐event analyses. In addition, the short median follow‐up may have led to under‐ascertainment of late local recurrences and increased uncertainty in long‐term local control estimates. Moreover, key oncologic variables—such as disease‐free interval and details of systemic therapy after cryoablation—could not be uniformly ascertained across the long enrollment period, which may have contributed to residual confounding in the survival analyses. Fourth, the enrollment period was long (2002–2017); thus, the potential influence of advances in medical technology and diagnostic imaging on treatment outcomes during this period cannot be excluded.

However, these real‐world data provide practical evidence supporting percutaneous cryoablation as a feasible and safe local treatment option for selected patients with pulmonary metastases from colorectal cancer. In particular, the ability to perform the procedure under local anesthesia may expand treatment options for patients who are not ideal candidates for surgery due to comorbidities or advanced age.

Our findings contribute clinically relevant information to support decision‐making in the multidisciplinary management of metastatic colorectal cancer.

## 5 Conclusion

This large single‐center series demonstrates that percutaneous cryoablation provides a safe and effective treatment option, with favorable long‐term local control and overall survival in patients with pulmonary metastases from colorectal cancer. Its safety and efficacy are comparable to those of other local therapies, while offering additional advantages such as repeatability, the ability to treat lesions adjacent to vital structures, and relatively low procedure‐related pain. Collectively, these findings support cryoablation as an important local treatment option for pulmonary metastases from colorectal cancer.

## Author Contributions

**Shun Yorimori:** data curation, formal analysis, investigation, visualization, and writing – original draft. **Kaoru Kaseda:** conceptualization, data curation, formal analysis, investigation, methodology, writing – review and editing, and supervision. **Yusuke Aoki:** resources. **Kosuke Sugino:** resources. **Takahiro Suzuki:** resources. **Yu Okubo:** resources. **Shigeki Suzuki:** resources. **Kyohei Masai:** resources. **Masashi Tamura:** methodology, investigation, and resources. **Masanori Inoue:** methodology, investigation, and resources. **Hideki Yashiro:** methodology, investigation, and resources. **Seishi Nakatsuka:** conceptualization, data curation, methodology, investigation, and resources. **Yoshikane Yamauchi:** conceptualization, data curation, investigation, and methodology. **Yotaro Izumi:** conceptualization, data curation, investigation, and methodology. **Masafumi Kawamura:** conceptualization. **Masahiro Jinzaki:** conceptualization and supervision. **Keisuke Asakura:** project administration and writing – review and editing.

## Funding

The authors have nothing to report.

## Consent

The requirement for informed consent was waived owing to the retrospective design.

## Conflicts of Interest

The authors declare no conflicts of interest.

## Supporting information


**Figure S1:** Area under the receiver operating characteristic curve for tumor diameter for predicting local recurrence after cryoablation. AUC, 0.600 (95% CI, 0.491–0.708), *p* = 0.071. Tumor diameter of 1.4 (arrow) as the hypothetical threshold yielded 51.6% sensitivity and 30.5% specificity. AUC: area under the receiver operating characteristic curve.
**Figure S2:** Primary local tumor control rates for all the treated tumors according to tumor diameter
**Figure S3:** Primary local tumor control rates for all the treated tumors according to tumor location.
**Figure S4:** (a) Primary local tumor control rates for all the treated tumors based on extrathoracic metastases before cryoablation. (b) Primary local tumor control rates for all the treated tumors based on the number of concomitantly treated tumors.
**Table S1:** Comparison of baseline tumor characteristics between central and noncentral tumors.

## Data Availability

The data that support the findings of this study are available from the corresponding author upon reasonable request.
