# Supplementary material for: Percutaneous Cryoablation Under Local Anesthesia for Pulmonary Metastases From Colorectal Cancer: Long‐Term Outcomes From a Single‐Institution Retrospective Cohort
Source: Cancer Rep (Hoboken). 2026 Apr 19;9(4):e70550. doi: 10.1002/cnr2.70550 (PMC13092425; doi:10.1002/cnr2.70550)
Supplement: Supplementary file 1 — Figure S1: Area under the receiver operating characteristic curve for tumor diameter for predicting local recurrence after cryoablation. AUC, 0.600 (95% CI, 0.491–0.708), p = 0.071. Tumor diameter of 1.4 (arrow) as the hypothetical threshold yielded 51.6% sensitivity and 30.5% specificity. AUC: area under the receiver operating characteristic curve. Figure S2: Primary local tumor control rates for all the treated tumors according to tumor diameter Figure S3: Primary local tumor control rates for all the treated tumors according to tumor location. Figure S4: (a) Primary local tumor control rates for all the treated tumors based on extrathoracic metastases before cryoablation. (b) Primary local tumor control rates for all the treated tumors based on the number of concomitantly treated tumors. Table S1: Comparison of baseline tumor characteristics between central and noncentral tumors. [file CNR2-9-e70550-s001.pdf]

Supplementary Information

Percutaneous cryoablation under local anesthesia for pulmonary metastases from colorectal cancer:
Long-term outcomes from a single-institution retrospective cohort

Shun Yorimori[1], Kaoru Kaseda[1*], Yusuke Aoki[1], Kosuke Sugino[1], Takahiro Suzuki[1], Yu Okubo[1], Shigeki Suzuki[1], Kyohei Masai[1], Masashi Tamura[2], Masanori Inoue[2], Hideki Yashiro[2], Seishi Nakatsuka[2], Yoshikane Yamauchi[1], Yotaro Izumi[1], Masafumi Kawamura[1], Masahiro Jinzaki[2], Keisuke Asakura[1]

Supplementary Figure S1

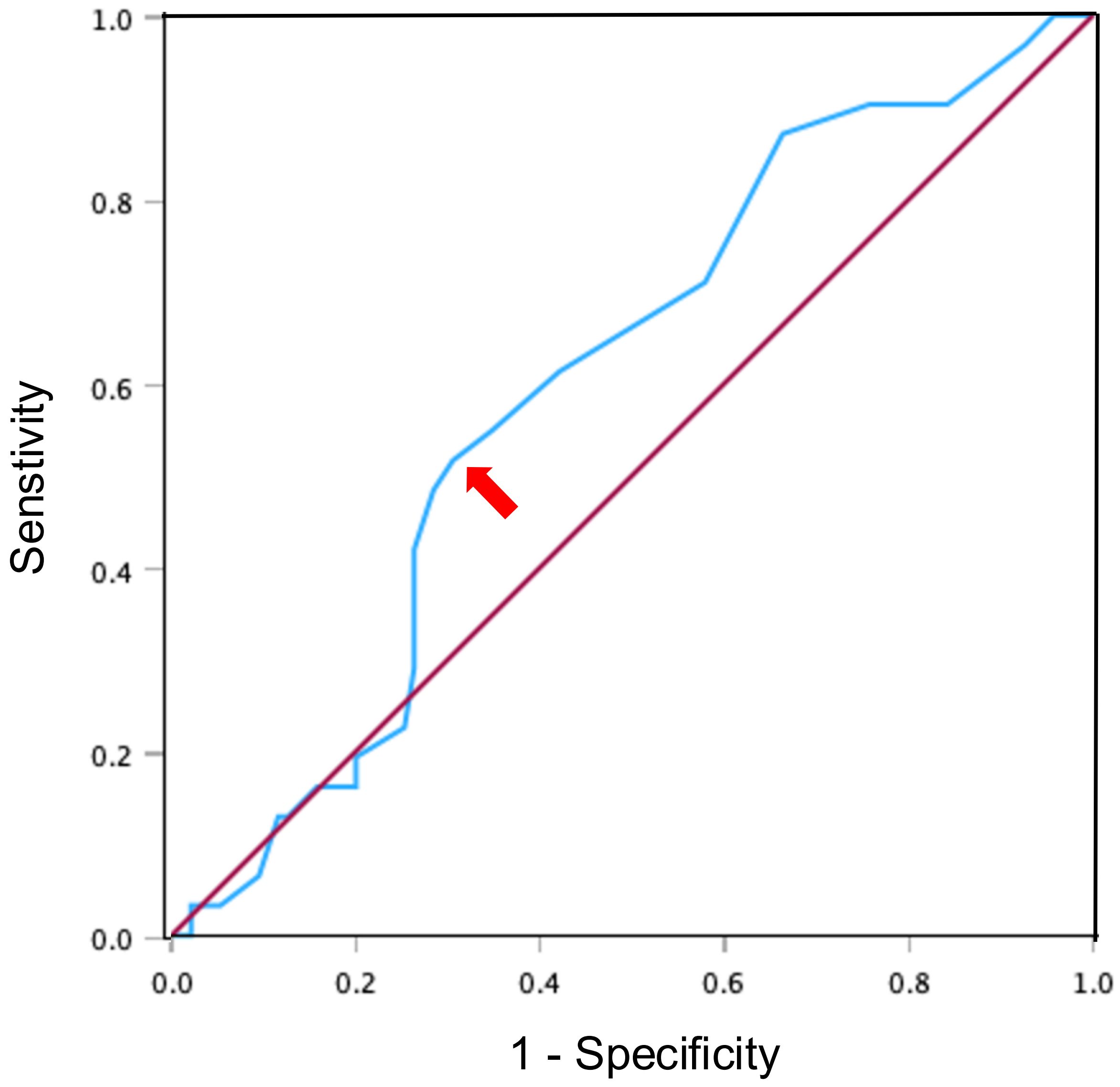

Supplementary Figure S1. Area under the receiver operating characteristic curve for tumor diameter for predicting local recurrence after cryoablation. AUC, 0.600 (95% CI, 0.491–0.708), P = 0.071. Tumor diameter of 1.4 (arrow) as the hypothetical threshold yielded 51.6% sensitivity and 30.5% specificity. AUC: area under the receiver operating characteristic curve.

Supplementary Figure S2

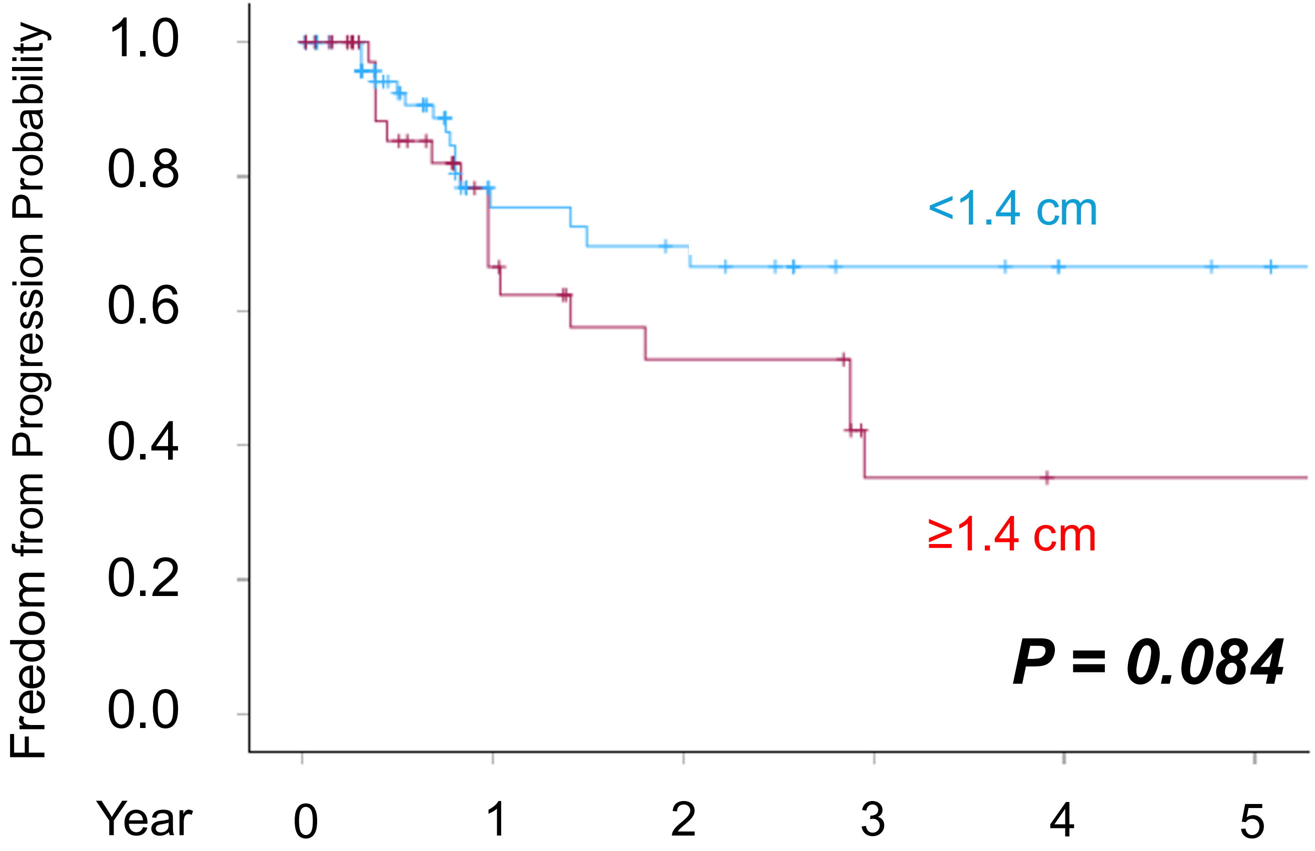

Tumors at risk

	0	1	2	3	4	5
<1.4 cm	81	26	23	16	13	12
≥1.4 cm	45	17	11	5	4	4

Supplementary Figure S2.

Primary local tumor control rates for all the treated tumors according to tumor diameter.

Supplementary Figure S3

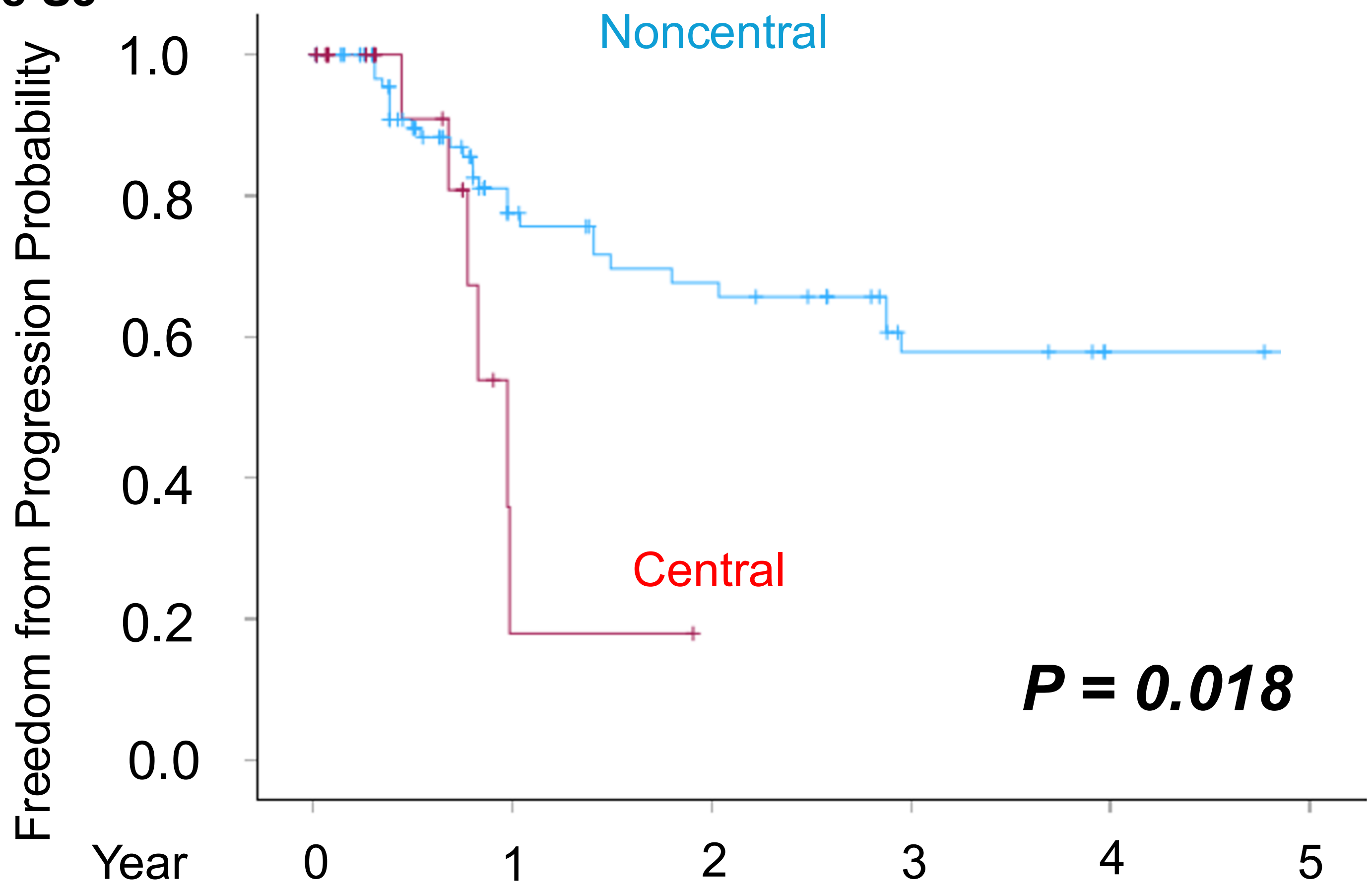

Tumors at risk

	0	1	2	3	4	5
Noncentral	99	42	34	21	17	16
Central	27	1	0	0	0	0

Supplementary Figure S3.

Primary local tumor control rates for all the treated tumors according to tumor location.

Supplementary Figure S4

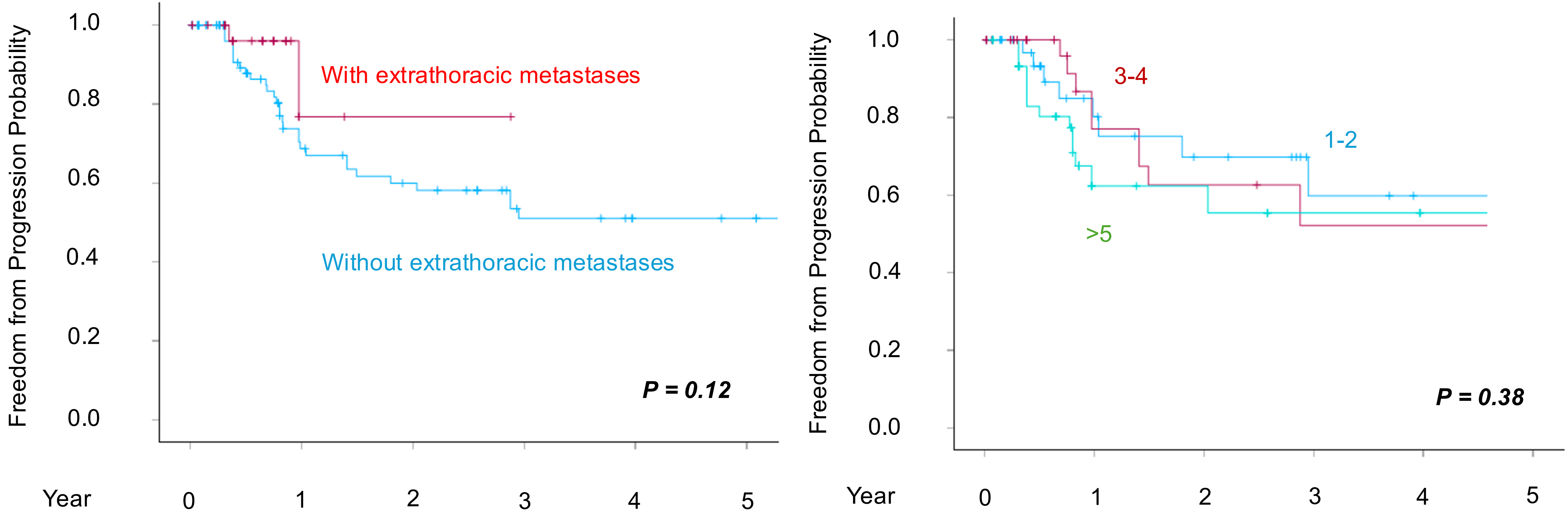

Tumors at risk

Without extrathoracic metastases	93	41	33	21	17	16
With extrathoracic metastases	33	2	1	0	0	0

Tumors at risk

1-2	36	17	12	6	4	3
3-4	42	16	13	10	10	10
>5	48	10	9	5	3	3

Supplementary Figure S4. (a) Primary local tumor control rates for all the treated tumors based on extrathoracic metastases before cryoablation. (b) Primary local tumor control rates for all the treated tumors based on the number of concomitantly treated tumors.

Supplementary Table S1

Characteristics	Central tumors	Noncentral tumors	p-value
N tumors	27	99	
Tumor diameter, cm (median, range)	1.2 (0.8-5.3)	1.0 (0.3-4.0)	0.8
Lobar distribution			0.55
Left superior lobe	8 (29.6%)	28 (28.3%)	
Left inferior lobe	2 (7.1%)	22 (22.2%)	
Right upper lobe	7 (25.9%)	21 (21.2%)	
Right middle lobe	2 (7.1%)	6 (6.1%)	
Right inferior lobe	8 (29.6%)	22 (22.2%)	
Number of tumors treated in the same session(median, range)	2 (1-6)	2 (1-7)	0.25

Supplementary Table S1.

Comparison of baseline tumor characteristics between central and noncentral tumors.
